# Evaluating the adhesive potential of the newly isolated bacterial strains in research exploitation of plant microbial interaction

**DOI:** 10.3389/fpls.2022.1004331

**Published:** 2022-10-21

**Authors:** Shifa Shaffique, Muhammad Imran, Shabir Hussain Wani, Muhamad Aqil Khan, Sang-Mo Kang, Arjun Adhikari, In-Jung Lee

**Affiliations:** ^1^ Department of Applied Biosciences, Kyungpook National University, Daegu, South Korea; ^2^ Mountain Research for Field Crops Khudwani, Sher-e Kashmir University of Agricultural Sciences and Technology of Kashmir, Srinagar, Jamu and Kashmir, India

**Keywords:** biofilm, motility, sem, hydrophobicity, bbf

## Abstract

Bacterial adhesion potential constitutes the transition of bacteria from the planktonic to the static phase by promoting biofilm formation, which plays a significant role in plant-microbial interaction in the agriculture industry. In present study, the adhesion potential of five soil-borne bacterial strains belonging to different genera was studied. All bacterial strains were capable of forming colonies and biofilms of different levels of firmness on polystyrene. Significant variation was observed in hydrophobicity and motility assays. Among the five bacterial strains (SH-6, SH-8, SH-9, SH-10, and SH-19), SH-19 had a strong hydrophobic force, while SH-10 showed the most hydrophilic property. SH-6 showed great variability in motility; SH-8 had a swimming diffusion diameter of 70 mm, which was three times higher than that of SH-19. In the motility assay, SH-9 and SH-10 showed diffusion diameters of approximately 22 mm and 55 mm, respectively. Furthermore, among the five strains, four are predominately electron donors and one is electron acceptors. Overall, positive correlation was observed among Lewis acid base properties, hydrophobicity, and biofilm forming ability. However, no correlation of motility with bacterial adhesion could be found in present experimental work. Scanning electron microscopy images confirmed the adhesion potential and biofilm ability within extra polymeric substances. Research on the role of adhesion in biofilm formation of bacteria isolated from plants is potentially conducive for developing strategies such as plant–microbial interaction to mitigate the abiotic stress.

## Introduction

**Figure 1:**
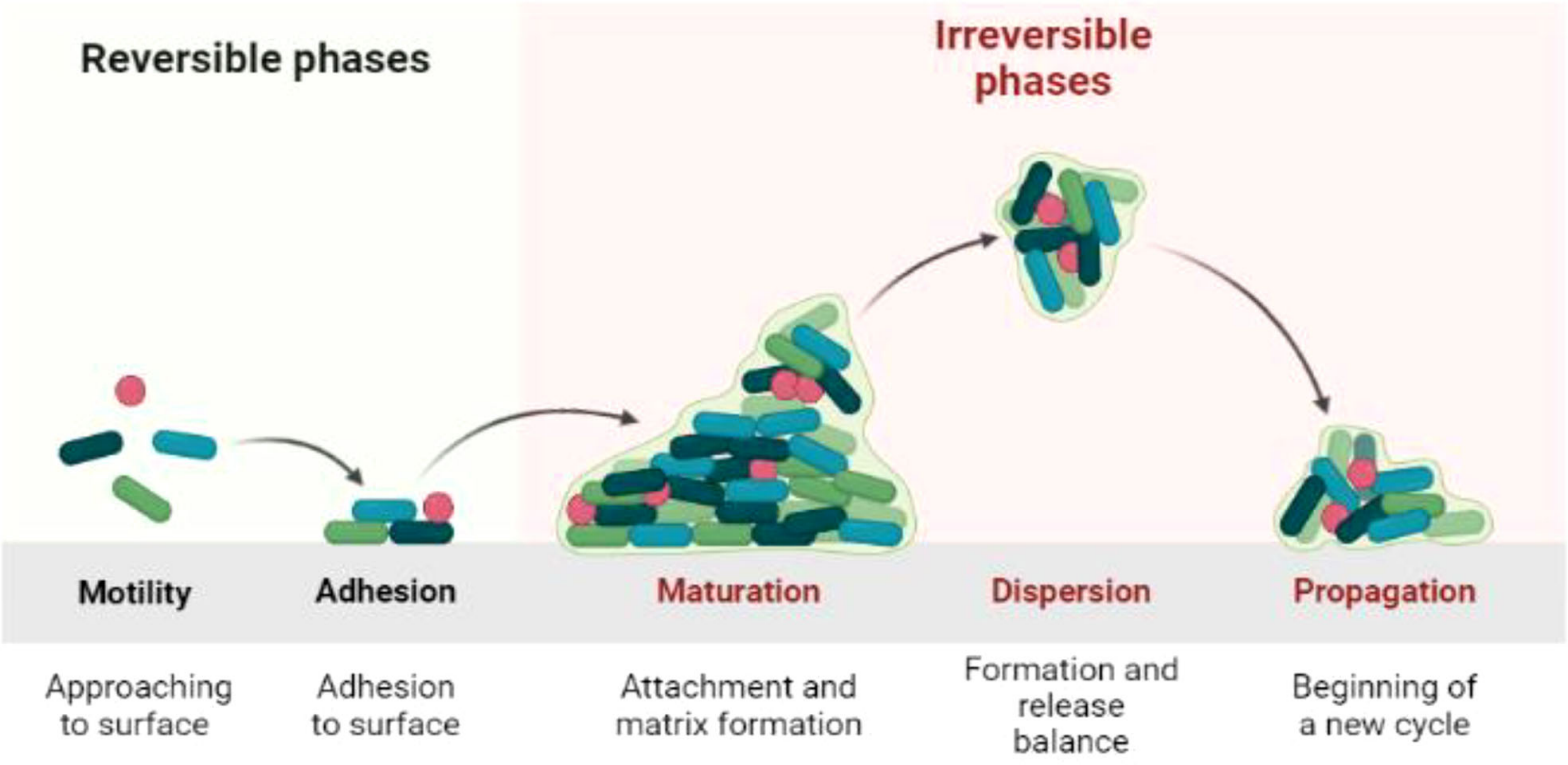
Biofilm life cycle of bacteria.

Biofilm is the product of microbial developmental procedure. It is a syntrophic relationship of the microorganism with any biotic or abiotic surface (Watnick and Kolter, 2000; Flemming and Wingender, 2010). The microbes become adherent to an extracellular polymeric substance. During interaction with the surface, microbes produce long chain exopolysaccharides and form a 3D coordinated functional community (O’toole et al., 2000; Lewandowski and Beyenal, 2019). The biofilm lifecycle of the microbes consists of a series of steps and events. The first step is attachment. The microbes are in the planktonic cell mode of biofilm formation with phenotype alteration by quorum sensing (QS) signaling pathway, and 90% of the cell biomass is produced in this phase (Rittmann and Mccarty, 1980; Tolker-Nielsen, 2015). Microbes attach to a surface using weak van der Waals forces. The QS signaling pathway is responsible for the formation of colonies and sub-colonies depending upon the temperature, p, and nutrient availability status. The colonization may extend from unicellular to multicellular cells, where microbes share traits related to nutrition, shelter etc. (Van Loosdrecht et al., 2002; Allison, 2003; Flemming et al., 2011). The bacterial life cycle can be categorized into four phases: log, lag, stationary, and decline phase. The lag phase is explained as the state where there is no proliferation of living bacteria as shown in the **Figure 1**. In the log phase, the bacteria start to grow rapidly, whereas, in the stationary phase, the death rate and production of bacteria are at a constant rate (Finkel, 2006; Saikia et al., 2022). Decline is the last phase where there are more dead bacterial cells than living (Andrade et al., 2006).The adhesion potential of bacteria can be observed from the log phase to the stationary phase (Swinnen et al., 2004; Jaishankar and Srivastava, 2017; Chevallereau et al., 2022).

After forming colonies, bacteria produce long chain polysaccharides that enclose the bacterial biofilm (BBF) as the cells mature. The life cycle of bacteria is completed in the biofilm, which then becomes ready for dispersion (Flemming et al., 2007; Renner and Weibel, 2011). The BBF disperses with the help of the enzymes dispersin B and deoxyribonuclease. The biofilms are unique owing to the differential expression of genes within these biofilms (Stoodley et al., 1999; Donlan, 2001; Teughels et al., 2006).

Biofilm forming microbes play a noteworthy role in biofertilization, mineralization, development, and maintenance of plant and soil fertility. Biofilms provide a biologically active metabolite exchange platform for plants and soil (Turhan et al., 2019; Kour et al., 2020; Jiang et al., 2021). They may form symbiotic, parasitic, or mutualistic relationships with the ecosystem. Microbes form colonies and biofilm on the leaves, roots, stem, rhizosphere, endosphere, rhizoplane, and phyllosphere of plants (Seneviratne et al., 2010; Seneviratne et al., 2011; Nayak et al., 2020). BBF affects growth attributes directly or indirectly *via* induced systemic resistance (ISR). Plants secret exudates that form the best surface for microbial attachment owing to high nutrient availability (Velmourougane et al., 2017; Kumar and Singh, 2020; Rheinheimer Dos Santos et al., 2020). Plant cell surface is the favorable site of attachment for bacterial establishment in tropical ecosystems (Bhat K and Bhat Panemangalore, 2022).

BBF is important in abiotic stress tolerance for providing an attachment site for bacteria and enhancing their potential to evolve during plant–microbial interaction (Zhang et al., 2020; Shaffique et al., 2022b). Microbes form dynamic interactions with plants and improve plant productivity, and physiological and molecular processes through a cascade of events such as production of osmolytes, organic acids, volatile compounds, siderophores, exopolysaccharides, and phytohormones and promotion of gene expression. Various recent studies have supported plant–microbial interaction in enhancing growth, development, and stress tolerance (Bhagat et al., 2021; Ciofu et al., 2022; Sachdev and Ansari, 2022). Scanning electron microscopy (SEM) is used to determine biofilm investigation. It provides information on the size, structure, location within the biofilm, and bacterial contact on extra polymeric substances (EPS) (Priester et al., 2007; El Abed et al., 2012).

Agriculture laboratories are commonly known for developing expedient technological properties in the production of beneficial microbes and their interaction with plants, but these microbes could also be involved in plant pathogenesis. It is well known that plant–microbial interactions occur through biofilms (Bednarek and Osbourn, 2009; Berg, 2009). However, only fewer studies have been accompanied on the adhesion potential of bacteria and their association with plants. For this reason, five protagonist bacterial isolates, namely, SH-6, SH-8, SH-9, SH-10, and SH-19, from plant-related sources were considered in the present experimental study.

The current study aim’s is to estimate the 1) adhesion potential of five bacterial isolates from plant-related sources, 2) characterize their cell properties including motility and surface hydrophobicity, 3) determine the correlation between adhesion potential and cell surface hydrophobicity, and 4) create SEM images to visualize the biofilm. The present study aims to enrich the speculative background of microbial biofilm formation and provide evidence on the adhesion potential for further research progress in plant–microbial interaction.

## Materials and methods

### Bacterial isolation

Seventy-three microbes originating from the rhizospheric soil of *Artemisia princeps* Pamp. were collected and isolated for further screening assays. The rhizospheric soil was collected from Pohang beach in South Korea, at an elevation of 9.9 m. The sample was preserved in a polyethylene bag in an icebox for transportation to the crop physiology lab, College of Applied Biosciences, Agriculture department, Kyungpook National University, South Korea. One gram of soil removed from the roots of the plants was mixed with 1 M saline solution, and the sample was retained in a shaker at 25°C, for 6 h. The sample was considered as a stock solution. From this stock solution, a serial dilution 10^-1^-10^-9^ was made for further analysis. Lysogeny broths were prepared, and aliquots of the serial dilutions were added. The plates were sealed with Parafilm and incubated for 24-72 h at 25°C ± 3°C. Immediately after the emergence of colonies, they were re-plated on LB plates until to obtain pure colonies. These colonies were identified by their size, shape, color, chain length, and growth pattern. The samples were then sent to Solgent Company for identification. The procedure followed is described as in published studies (Chung et al., 2005; Rashid et al., 2012).

### Molecular identification and phylogenetic analysis

#### Inocula preparation

The bacterial strains were individually cultured at 37°C for 18 h in 5 mL of LB. The cells were then harvested by centrifugation at 5000 rpm for 5 min at 4°C. The supernatant was removed, and the cells were washed thrice with saline phosphate buffer solution (pH 7.2) and resuspended in saline phosphate buffer solution to maintain a constant pH. The final inoculum of 10^3^ CFU/mL was prepared as described previously (Ramirez-Chavarin et al., 2013; Li et al., 2020).

#### Biofilm assay

For each inoculum of 150 µL, 20 µL from freshly prepared sample and 130 µL from pre-prepared inoculum were combined in a 96-well polystyrene microplate. The samples were incubated at 25-30°C for 5 days. Freshly prepared autoclaved media devoid of bacteria were used as the negative control. After 5 days, the microplate was rinsed three times with 0.1% saline phosphate buffer solution and air dried for 30 min. Then, 100 µL crystal violet stain was added and washed three times with phosphate buffer solution. At the end of the procedure, 0.2 mL of 95% ethanol was added to the plates, and the absorbance was measured at 570 nm. The procedure was followed as described previously (Coffey and Anderson, 2014; Shukla and Rao, 2017).

#### Hydrophobicity assay

Equal amounts of solvents (chloroform, ethyl acetate, and xylene) were mixed with the bacterial suspension to formulate a two-phase system. The solutions were vortexed for 4 minutes and placed at room temperature for 30 min to allow the hydrocarbon phase to increase. The optical density (OD) was calculated at 600 nm before and after the adhesion. The hydrophobicity was calculated using the following equation:


Hydrophobicity %=[( A0−A1)/A0]×100

where A0 is considered as the initial optical density, and A1 is the final optical density of the aqueous phase, as described previously (Rahman et al., 2008; Chao et al., 2014).

#### Motility assay

The motility assay (swimming and swarming) was performed on soft agar plates. The plates for the swimming assay were prepared by mixing 2.5 g/L glucose, 5 g/L sodium chloride, 10 g/L tryptone, and 0.3% agar, while those for the swarming assay contained, 0.5 g/L glucose, 25 g/L Luria-Bertani and 0.5% agar. Two microliter aliquots of each isolates were mottled onto the soft-agar plates surface and then incubated at 25-30°C for 15 min to ensure better absorption. The diameter (mm) of each strain was measured in order to estimate the motility (Morales-Soto et al., 2015; Sun et al., 2018).

#### SEM microscopy

Bacterial isolates were allowed to grow in the LB plates for 48 h after which pure isolates together with the media were cut (W × D × H, 5 × 2 × 5 mm) and placed into Eppendorf tubes. Then, 0.1 M solution of phosphate buffer (pH 7.3) was added to the tubes and washed three times. The samples were precleared with 2.5% glutaraldehyde solution for 24 h at 4°C. The fixation sample was placed into 0.1 M sodium phosphate buffer solution (pH 7.3) and washed again with 0.1 M sodium phosphate buffer solution. Then, 1% osmium tetroxide in 0.1 M cacodylate buffer was added to the sample for 90 min and washed with sodium phosphate buffer for 40 min. After washing, the samples were dehydrated with ethanol. Excess water was removed using a critical point device, carbon tapes were attached, and the samples were observed under a scanning electron microscope (Hitachi-s-3500N), as previously described (Chen et al., 2022; Kuzmina et al., 2022).

#### Statistical analysis

All the experiments were repeated five times. The GraphPad Prism software (version 5.8) was used to perform statistical analysis. The mean values P ≤ 0.05 were considered significant using Duncan’s multiple range test (DMRT) in SAS (version 9.1) to find out the statistical analysis.

## Results

### Biofilm assay

**Figure 2:**
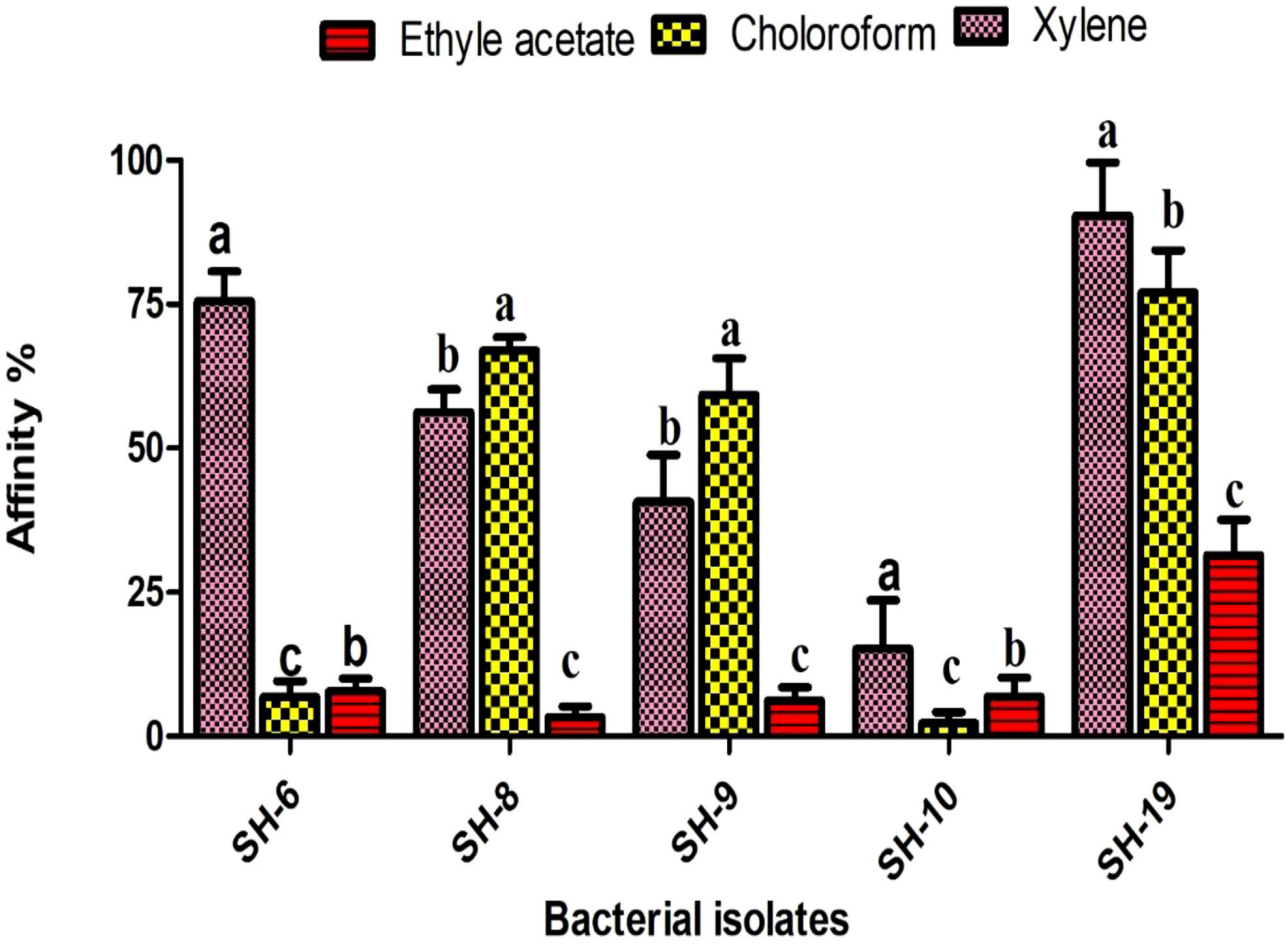
Information of the five selected isolates and their affinity for different solvents. The letter on each bar is the significant different between treatments at P ≤ 0.05. The error bar represents the standard error among the replicates.

Xylene is considered as polar. All isolates showing strong affinity toward xylene were labeled as hydrophobic. SH-10 had the lowest affinity toward xylene, at less than 20%, which shows the strongest electron acceptor ability. Isolates showing moderate affinity toward chloroform were electron donors (**Figure 2**).

### Cell-surface characteristic assay

**Figure 3:**
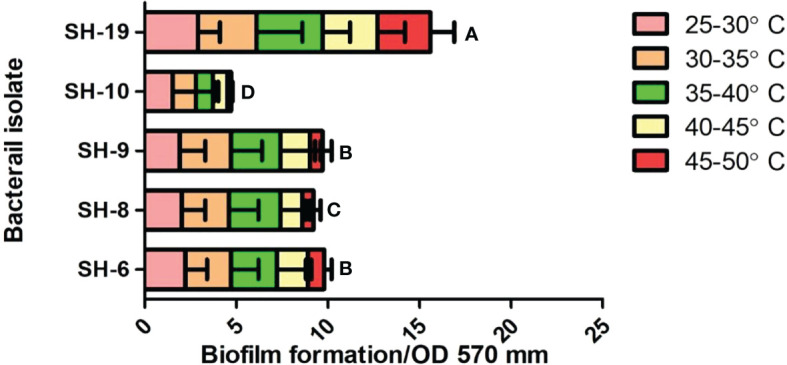
Biofilm information by the five selected isolates on 96-well polystyrene microplates under 22–50°C for 24 h. The letter on each bar is the significant different between treatments at P ≤ 0.05. The error bar represents the standard error among the replicates.

All bacterial isolates possessed adhesion potential for the 96-well plate with different levels of firmness. However, SH-19 showed excellent biofilm formation compared to that by the other four bacterial isolates. Thus, SH-6, SH-8, and SH-9 are moderate biofilm-producing bacterial isolates, while SH-10 had the lowest biofilm-forming ability (**Figure 3**).

### Observation of bacterial biofilm by SEM

**Figure 4:**
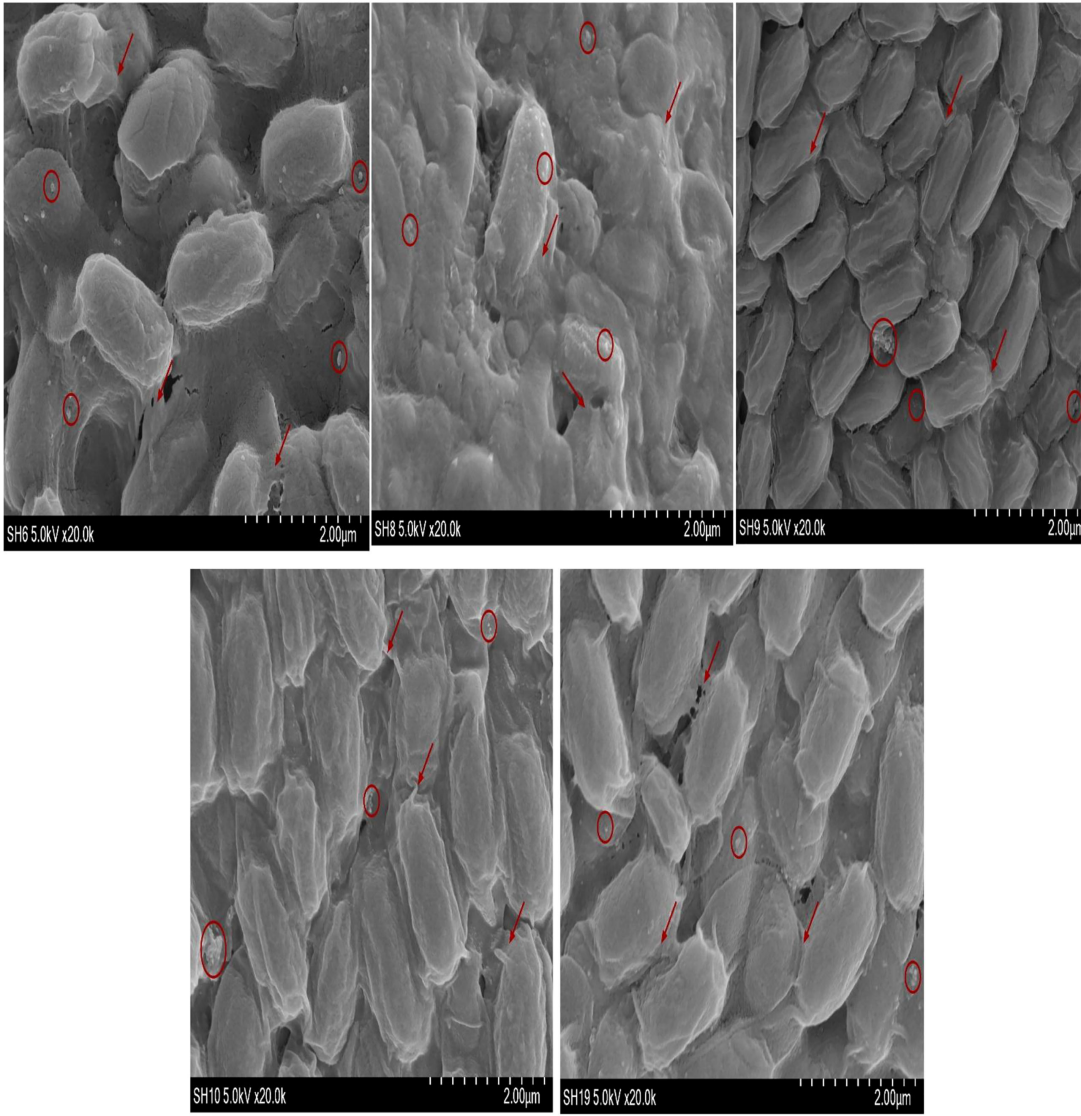
Scanning electron microscopy images of five bacterial isolates (scale bar, 2.00 µm).

The SEM shown in **Figure 4** confirms the presence of biofilm whereas the ↗○ represents the location and position of exopolysacchrides

## Discussion

BBF formation plays an significant role in plant–microbial interaction in sustainable agriculture industry (Ramey et al., 2004; Bogino et al., 2013). This study involved 5 protagonist soil-borne bacterial isolates for biofilm assay. The optimal temperature of each bacterial isolate varied with an average of 25°C and which was considered as the optimal temperature.

The short and long-term biofilms were evaluated in a time-dependent manner by regulating the incubation time by 24-72 h. The results exhibited that all bacterial isolates were capable to attach on the polystyrene surface with some variation which exposed the isolates belongs to different species. SH-19 exhibited the best adhesion ability regardless of time and temperature, while a moderate amount of biofilm formation was displayed by SH-10, SH-8, SH-6, and SH-9. All strains formed considerable amounts of biofilm at 25-30°C after 24 h. However, for 72 h incubation, a higher temperature was most appropriate for bacterial cell adhesion. The entire different behaviors shown by SH-9 in comparison with SH-19 confirmed that all isolates from a single source do not necessarily have the same characteristics.

SH-19 is an extremophile that can exist at extreme temperatures and produce and maintain biofilms at 50°C. However, the surface of the 96-well polystyrene plate that was used for the present *in vitro* experimental study is significantly different from that of the plant epidermis, and thus, the activities of related signaling pathways could be limited. Biofilm formation was temperature-dependent as shown in **Figure 3**. At 24 h, four of the five bacterial isolates displayed a higher attachment at above 30°C than 40°C; after 72 h, only SH-19 showed significant biofilm formation. A further increase in temperature to 50°C resulted in the formation of considerable amount of biofilm by SH-19. This is a well-known phenomenon and occurs probably owing to the diversity of optimal temperatures of the isolates (Rozanov et al., 2021).

Massive cell density in culture media possibly results in providing an assistance for communication of bacteria, thus, improving the bacterial attachment. Additionally, less biofilm formation by SH-9, SH-6, SH-8, and SH-10 at 50°C after 24 h is probably owing to biofilm dispersal at the end of the incubation periods. The outcomes of study also indicated that temperature and time influenced biofilm formation.

In one complete biofilm development cycle, dispersion is the final step, in which cells detach from the established biofilm at high temperatures (Rumbaugh and Sauer, 2020). After 72 h, the restricted biofilm may be owing to limited nutrition perfusion. The period during which bacteria show impaired adhesion may be owing to the dispersion phase. At 25-30°C, mostly the isolates grow fast over time and form mature biofilms. At 35°C, after 24 h, there is a decreased amount of biofilm by 72 h. This partially proved that higher temperature was preferable for most bacteria. Attention must be given to the existence of particularity in biofilm formation.

Cell adhesion entails contact with the interacting surface of the bacterial cell envelope. Motile properties are important at the docking phase when two objects meet (Ottemann and Miller, 1997). Swimming and swarming motility was investigated in the present study. Swimming is related to the motile potential of individual bacterium, whereas swarming is related to colony formation (Kearns, 2010; Servant et al., 2015; Be’er and Ariel, 2019). SH-6 and SH-8 showed considerable motility and developed large diffusion circles with diameters more than 70 mm, three times larger than those of SH-19 and SH-10.

SH-9 exhibited moderate swimming capacity with a diameter of 22 cm. A lower disparity level was found among the isolates in the swarming assay. The five bacterial isolates showed reserved swarming ability compared to their swimming potential. These results suggest that fluidity of the medium may influence the diffusion of bacterial isolates.

Flowing states might favor plant–microbe interaction and subsequent development of biofilm. Bacterial motility is flagella dependent. Swimming and swarming potential of microbes play important role in their adhesion and biofilm formation (Daniels et al., 2004; Venieraki et al., 2016).

Because different strains of different species possess a diverse range of flagellar numbers, great variations are noted in motility. SH-10 showed a diffusion circle of 55 mm with remarkable motility but were not strong in biofilm formation. Furthermore, no correlation was observed between biofilm formation and motility of the isolates as shown in **Table 1**.

**Table 1:** Bacterial strain, motility and NCBI gene accession link.

Strain	Origin	Motilitymm	Gene accession number and NCBI Data base
SH-6	Plant	70 mm	https://www.ncbi.nlm.nih.gov/nuccore/OM757882.1
SH-8	Plant	68.9 mm	https://www.ncbi.nlm.nih.gov/nuccore/OM535901
SH-9	Plant	23.2 mm	https://www.ncbi.nlm.nih.gov/nuccore/On753949
SH-10	Plant	22.8 mm	https://www.ncbi.nlm.nih.gov/nuccore/OP175947
SH-19	Plant	55 mm	https://www.ncbi.nlm.nih.gov/nuccore/On935600

The function of flagella cannot be ignored. Studies using video microscopy have shown that flagella help bacteria reach the proximate surface, but in the present study, a partial positive correlation was observed.

The surface hydrophobicity is determined by xylene, a polar solvent. High affinity to xylene was noticed in SH-19 and SH-8, representing hydrophobicity, while SH-10 showed hydrophilicity, under 20%, toward xylene.

SH-6 and SH-10 showed competitor of hydrophobicity. Various recent studies have suggested that hydrophobic characteristics enhance biofilm formation potential. This is in line with the results of the present study in which we show a positive correlation and that hydrophobicity enhances biofilm formation.

Lewis acid base properties were also found by using two other solvents, chloroform and ethyl acetate. SH-10 showed strong affinity toward chloroform and ethyl acetate implying electron acceptor capacity. SH-6 showed almost equal affinity toward ethyl acetate and chloroform, which is predominately an electron donor and weak electron acceptor. The other bacterial isolates showed better affinity to chloroform than ethyl acetate representing their greater ability to donate electrons.

The bacterial isolates with electron donor capacity attached easily to the surface of the 96-well polystyrene plate. A positive correlation was noted between hydrophobicity and biofilm formation. Thus, Lewis acid base interaction shows an energetic role in biofilm formation.

SEM utilizes the high-power energy of electron beams to visualize the properties of bacterial cells such as their size, shape, homogeneity, and topological information. Additionally, it provides useful information about biofilm life cycle, from attachment to dispersion (Baldotto and Olivares, 2008; Nongkhlaw and Joshi, 2014). SEM also displayed the observation of bacterial adhesion properties to a surface and their ability to form biofilms. Bacterial adhesion to biofilm formation is the condition in which bacteria attach and adhere firmly to a surface and completes one life cycle (El Abed et al., 2012; Relucenti et al., 2021).

**Figure 5:**
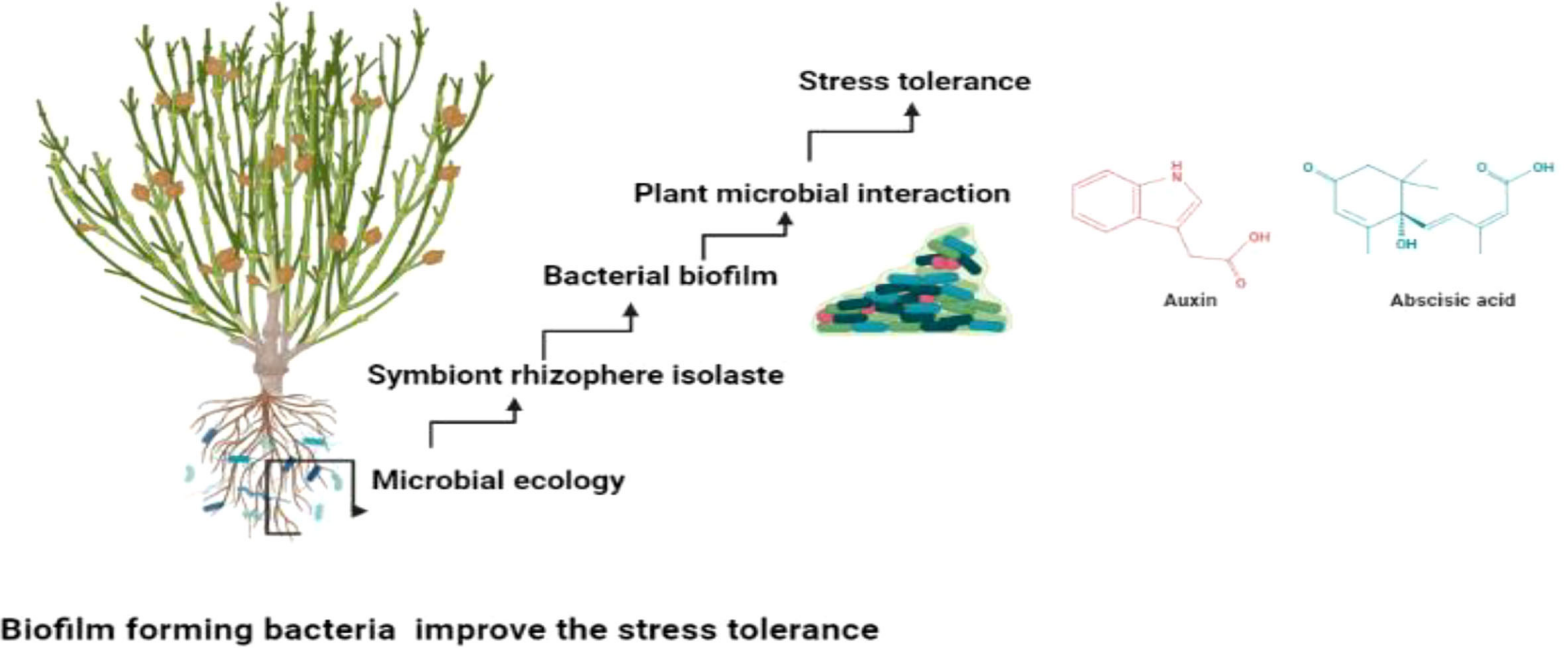
Biofilm producing isolates mitigate stress tolerance through plant microbial interaction.

It is important to estimate the adhesion potential of the bacterial isolates because adhesion potential of bacteria provides the transition from planktonic phase to static phase (Singh et al., 2011; Xu and Siedlecki, 2022). The transition of microbes from one phase to another phase is responsible for the development of biofilm. Adhesion is the first step of biofilms which is liable for the docking and locking phase of the biofilm (Chu et al., 2022; Prabu et al., 2022). Adhesion potential has been quantified using a number of automatic tools such as by measuring the production of bacteria in Congo red assay or under a conventional scanning microscope (Taj et al., 2012). SEM is considered as the gold standard to identify biofilm under different resolutions. Bacterial strains are also visualized by SEM. This is an advanced microbiology technique that can measure bacterial cell attachment and provide clear images of bacterial flagellum (Moreira et al., 2012). High-magnification SEM of SH-6 showed small spherical structures and numerous tubular projections on the surface of the growing vegetative cells. The bacterial architecture of SH-8 showed the presence of spherical structure trapped in the exopolymeric substances (**Figures 4**, **5**), while that of SH-9 and SH-10 showed a mat-like structure. SH-19 were spherical bacteria. All the bacteria showed the presence of exopolysaccharides in which the bacteria were trapped. Bacteria interact with plant cells in diverse ways (Shaffique et al., 2022c). The main feature of this interaction is to make colonies, in which the microbes adhere to plant cells as individual cells or in the form of clusters (Morris and Monier, 2003; Tshikantwa et al., 2018). The adherent bacterial populations, defined as biofilms, display structural alignments of several magnitudes. Each plant part has distinguishing levels of saturation such as nutrient availability and cellular chemistry, which affects the formation of biofilm. It is important to evaluate the adhesion potential to measure plant–microbial interaction, which is important in the mitigation of abiotic stress. The adhesive biofilm ability of the bacteria provides the platform to colonies on plant cells with aid of self-producing matrix (EPS) to provide the protective environment against various abiotic stress such as drought, salinity and heat stress etc. (Sharma et al., 2021; Shaffique et al., 2022a).

Beneficial microbes facilitates the mutualistic interaction which is Important in providing the plant microbial interaction. The plant microbial interaction augments the production of the metabolites which improve the plant productivity (Ben Zineb et al., 2022; Mafa‐Attoye et al., 2022). Several studies suggested that biofilm producing bacteria’s are helpful in not only mitigation of abiotic stress but also prevent phytopathogen (Chandwani and Amaresan, 2022; Singh and Chauhan, 2022). They produce the exopolysacchrides which gives the adhesion potential and facilitates the plant microbial interaction (Kant, 2022; Kapadia et al., 2022). They play imperative role in neutralizing the abiotic stress and improving crop yield and quality (Admassie et al., 2022; Siddique et al., 2022) as shown in **Figure 5**.

## Conclusion and future prospective

We concluded that the five soil-borne bacterial isolates were capable of colonization on the 96-well polystyrene plate surface. Cell surface hydrophobicity was positively correlated with biofilm formation, whereas swimming and swarming were negatively correlated with biofilm formation. However, further research on larger scale is needed to clarify this. The biofilm architecture of bacterial isolates was observed by SEM. The results revealed that bacteria were trapped in the exopolymeric substances. The biofilm-forming bacterial isolates strongly influence plant–microbial interaction to mitigate the abiotic stress. Ongoing studies on newly bacterial isolates will provide the new experimental frame work to mitigate the stress and helps in providing the sustainable agronomy.

## Data availability statement

The original contributions presented in the study are included in the article/supplementary materials, further inquiries can be directed to the corresponding author/s.

## Author contributions

SS write the original draft paper did the experiment and formal analysis as a part of her dissertation. MI and MAK did the software analysis. S-MK and AA did the graphical designing. I-JL did the supervision, provide funding and validate the results. SW did the critical review editing. All authors contributed to the article and approved the submitted version.

## Funding

National Research Foundation(NRF) grant funded by the korea goverment(MIST)(No.2022R1A2C1008993).

## Conflict of interest

The authors declare that the research was conducted in the absence of any commercial or financial relationships that could be construed as a potential conflict of interest.

## Publisher’s note

All claims expressed in this article are solely those of the authors and do not necessarily represent those of their affiliated organizations, or those of the publisher, the editors and the reviewers. Any product that may be evaluated in this article, or claim that may be made by its manufacturer, is not guaranteed or endorsed by the publisher.

## Abbreviations

SEM, Scanning electron microscope; EPS, Exopolysacchrides; BBF, Bacterial biofilm; OD, Optical density; QS, Quorum sensing; CFU, Colony forming unit.
